# Frontal Sinus Endoscopy in Combined Approach Frontal Sinusotomy for Post-COVID-19 Mucormycosis

**DOI:** 10.7759/cureus.95604

**Published:** 2025-10-28

**Authors:** Sangeetha T Sachithanandam

**Affiliations:** 1 Otolaryngology-Head and Neck Surgery, Employees’ State Insurance Corporation (ESIC) Medical College and Hospital, Chennai, IND

**Keywords:** combined approach, endoscopic, external, frontal sinus endoscopy, mucormycosis

## Abstract

Introduction

Sinonasal mucormycosis is a fulminant, life-threatening, invasive fungal infection that emerged as an epidemic during the second wave of the coronavirus disease 2019 (COVID-19) pandemic in developing countries such as India. The objective of this study is to evaluate the role of frontal sinus endoscopy, to assess the disease extent in invasive frontal sinus mucormycosis, and to study the effectiveness of a combined approach involving endoscopic and external frontal sinusotomy among post-COVID-19 invasive sinonasal mucormycosis involving frontal sinuses.

Methods

This retrospective observational study included 11 patients with post-COVID-19 invasive sinonasal mucormycosis involving unilateral or bilateral frontal sinuses admitted to a quaternary care center from March 2021 to March 2022. Early diagnosis with classic nasal endoscopic findings and prompt surgical intervention with endoscopic sinonasal debridement combined with external frontal sinusotomy were performed for all 11 cases. Frontal sinus endoscopic findings are documented. The patients were followed up for a minimum period of one year, and the results are presented.

Results

Frontal sinus endoscopy revealed necrotic black frontal sinus mucosa with fungal growth (9%); pale frontal sinus mucosa studded with necrotic black spots (18%); pale frontal sinus mucosa with fungal debris (27%); inflamed frontal sinus mucosa with pus, fungal debris (18%), and granulations (18%); and prolapsed dura into the frontal sinus due to posterior frontal sinus wall erosion (9%). Six (55%) out of 11 patients recovered and were declared cured from the disease. Four (36%) out of 11 patients died within the first week of stage 1 surgical debridement. One patient (9%) was lost to follow-up after eight weeks of stage 1 surgical debridement. The combined approach involving endoscopic and external frontal sinusotomy provided complete local surgical clearance up to the lateral corners and the walls of the frontal sinuses, which are nasal endoscopically inaccessible.

Conclusion

Frontal sinus endoscopic findings explain the necessity of combining external with endoscopic approaches for frontal sinus involvement in invasive mucormycosis, which is otherwise inaccessible. The endoscopic findings in invasive frontal mucormycosis are documented in this article for the first time, which add a significant input to the existing medical literature.

## Introduction

The mucormycosis of the paranasal sinuses is a life-threatening, debilitating, fatal fungal disease typically affecting immunocompromised individuals. Until 2019, the diagnosis of extensive sinonasal mucormycosis was rare and sporadically reported in patients with severe immunodeficiency states such as diabetes mellitus; hematological malignancies such as leukemia, lymphoma, multiple myeloma, and solid organ transplants; and retrovirus infections such as acquired immunodeficiency syndrome (AIDS). Ever since 2019, when the severe acute respiratory syndrome coronavirus 2 (SARS-CoV-2) caused the coronavirus disease 2019 (COVID-19) pandemic, sinonasal symptoms are strongly associated with the disease. The sudden increase in the incidence of the fatal mucormycosis in the paranasal sinuses among the post-COVID-19 patients suggests a strong, deadly association between the two [1].

With the reported mortality rates of 50%-80% from this rapidly progressing invasive sinonasal disease to intraorbital and intracranial extension, early diagnosis and management carry utmost significance to improve the survival rates [2]. Extensive surgical debridement aiming for complete local disease clearance with the multidisciplinary management of associated comorbidities is crucial to achieve good clinical outcomes [3]. We present our experience with 11 patients with post-COVID-19 invasive sinonasal mucormycosis with frontal sinus involvement managed with extensive endoscopic sinonasal surgical debridement combined with external frontal sinusotomy for the frontal sinuses, aiming for complete disease clearance in the endoscopically inaccessible areas of the frontal sinuses documented by frontal sinus endoscopy. The objective of this study is to evaluate the role of frontal sinus endoscopy to assess the disease extent in invasive frontal sinus mucormycosis and to study the effectiveness of a combined approach involving endoscopic and external frontal sinusotomy among post-COVID-19 invasive sinonasal mucormycosis involving frontal sinuses.

## Materials and methods

This retrospective observational study was conducted in the department of otorhinolaryngology in Gleneagles Global Health City, Chennai, India, from March 2021 to March 2022. This study was approved by the Institutional Ethics Committee for Biomedical and Health Research of Gleneagles Global Health City, Chennai (protocol ID: BHMR/2024/0105). Waiver of consent was obtained. All the patients in post-COVID-19 state with sinonasal symptoms presenting as outpatients or admitted to the COVID-19 isolation unit/intensive care unit were subjected to diagnostic nasal endoscopy and computed tomography (CT) of paranasal sinuses to rule out sinonasal mucormycosis during the second wave of COVID-19, as there was a high degree of suspicion due to the sudden surge in the numbers of post-COVID-19 mucormycosis. Eleven patients with post-COVID-19 invasive fungal sinonasal mucormycosis with significant involvement of unilateral or bilateral frontal sinuses were included in the study. Sinonasal mucormycosis without frontal sinus involvement or only frontal recess involvement was excluded from the study.

**Figure 1:**
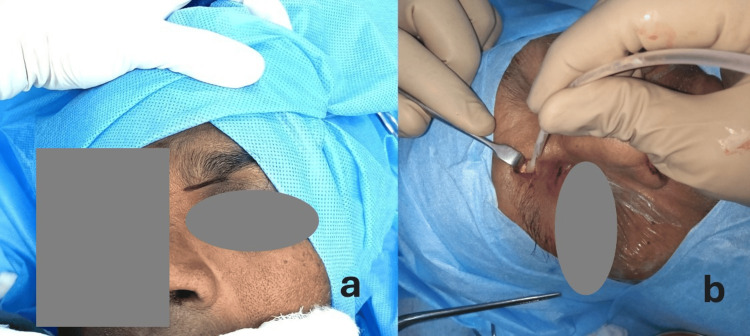
External frontal approach (a) External frontal approach: skin incision. (b) Fungal debris cleared off from the frontal sinus

A detailed history with multidisciplinary assessment involving ENT, ophthalmology, dental, neurology, neurosurgery, general physician, and infectious diseases physician was done. Diagnostic nasal endoscopy was done; swabs for fungal smear, aerobic culture and tissue for histopathology, and fungal identification were sent. All patients were subjected to CT and MRI of paranasal sinuses, orbit, and brain with or without contrast as per their renal parameters. Diagnosis was made on their classic endoscopic findings without waiting for tissue biopsy or culture reports, as immediate surgical debridement is necessary to halt the disease progression, in which failing means high rates of mortality and morbidity. All the patients were mobilized for surgery in the first 24-48 hours of clinical diagnosis as per their anesthetic fitness state. Bilateral endoscopic sinonasal surgical debridement involving endoscopic frontal sinusotomy (Draf IIa: fronto-ethmoid cell clearance from lamina papyracea to middle turbinate) combined with external frontal sinusotomy was done for all 11 patients as the first-stage surgery. An external frontal approach was made through a 1.5 cm medial eyebrow modified Lynch Howarth skin incision, the orbicularis oculi muscle was dissected, and a 1 cm-width frontal sinusotomy was drilled and further extended up to 1.5 cm as required, just adequate for the introduction of an angled endoscope and an instrument (Figure 1a). Local disease clearance was done, and tissue samples were sent for pathology and microbiological examination (Figure 1b). The primary closure of the frontal skin incision was done. All the patients received intravenous (IV) liposomal amphotericin B for 2-14 days based on their tolerance and comorbidities and were managed medically by the concerned team of physicians.

Revision endoscopic sinonasal debridement with orbital exenteration and varied levels of maxillectomies were done as second-stage surgeries as required. Postoperative nasal endoscopic follow-up was scheduled every fifth day for the first four weeks, fortnightly for the second month, and monthly from three to 12 months. CT of paranasal sinuses with the brain was done monthly for the first three months, and then, imaging was done as required based on the clinical symptoms, signs, and endoscopic findings. Maintenance therapy with oral posaconazole/isavuconazole for 6-12 weeks was administered with regular blood parameters such as complete blood count, renal function test, liver function test, serum electrolytes, and ECG monitored by the team of physicians. The patients were followed up for a period of one year, and the results were documented. The patients were declared cured nearing the end of the follow-up period after clearing two consecutive nasal endoscopies, and imaging at three-month intervals reveals no progression or new-onset disease. Data regarding the clinical presentations, investigations, treatment methods, outcome of the treatment, and follow-up details were collected, organized, and presented.

## Results

Patients’ data are depicted in Table 1. Among the 11 patients, three (27%) were women, and eight (73%) were men. Two (18%) patients were in the 45-50 years group, five (46%) patients in the 51-60 years, and four (36%) patients in the 61-70 years. Eight (73%) out of 11 patients were diabetic; among the eight diabetic patients, five (46%) had coexisting comorbidities such as coronary artery disease, cardiomyopathy, alcoholic liver cirrhosis, chronic kidney disease, and chronic obstructive pulmonary disease. The interval between COVID-19 and sinonasal symptoms ranges from six to 40 days, with a mean of 24 days. Five (46%) out of 11 patients had steroid therapy, though only two (18%) of them had noninvasive ventilation.

**Table 1:** Patients’ data M, male; F, female; CAD, coronary artery disease; CLD, chronic liver disease; CKD, chronic kidney disease; COPD, chronic obstructive pulmonary disease; CABG, coronary artery bypass graft; ESS, endoscopic sinus surgery; IV, intravenous; POD, postoperative day; COVID-19, coronavirus disease 2019

Parameters	Case 1	Case 2	Case 3	Case 4	Case 5	Case 6	Case 7	Case 8	Case 9	Case 10	Case 11
Age/sex	60/F	51/M	48/F	53/M	64/M	67/M	62/M	55/M	58/M	48/F	68/M
Comorbidities	Diabetic/CAD	Nil	Diabetic	Hypertension	Diabetic	Diabetic/CAD/CLD/CKD on hemodialysis	Diabetic/COPD/hypertension	Nil	Diabetic/CAD	Diabetic	Diabetic/COPD/CAD-post-CABG cardiomyopathy
Interval between COVID-19 and sinonasal symptoms in days	25 days	32 days	7 days	32 days	6 days	7 days	35 days	40 days	30 days	25 days	30 days
Steroid therapy	Yes. Noninvasive ventilation	Nil	Nil	Nil	Nil	Yes	Yes	Nil	Yes	Nil	Yes. Noninvasive ventilation
Clinical presentation	Right headache, periorbital pain and swelling, anosmia, and nasal block and discharge	Right headache, periorbital pain and swelling, and progressive loss of vision	Left headache, periorbital pain and swelling, anosmia, left cheek pain and swelling, proptosis, chemosis, ophthalmoplegia, left loss of vision, dental pain, and palatal eschar, left side	Bifrontal headache, anosmia, ocular pain, and nasal block and discharge	Left headache, periorbital pain and swelling, anosmia, and nose block and nasal discharge	Left headache, periorbital pain and swelling, left cheek pain and swelling, anosmia, proptosis, chemosis, ophthalmoplegia, and left progressive loss of vision	Left headache, periorbital pain and swelling, left cheek pain and swelling, dental pain, and left palatal eschar	Left cheek pain and swelling, ocular pain, and nasal block and discharge	Left headache, periorbital pain and swelling, left cheek pain and swelling, proptosis, chemosis, ophthalmoplegia, and left progressive loss of vision	Left headache, periorbital pain and swelling, left cheek pain and swelling, nasal block and discharge, and left dental pain	Bilateral headache, frontal swelling, and nasal block and discharge
Nasal endoscopy	Black eschar on the right middle turbinate and lateral wall	Black eschar on the right middle turbinate and lateral wall. Eroded lamina papyracea intraoperatively	Black eschar on the left middle turbinate and lateral nasal wall. Necrotic lamina papyracea intraoperatively (Figure 2)	Pale mucosa over the anterosuperior septum, bilateral cribriform plate, right medial aspect of the middle turbinate, and pus on the right middle meatus	Black eschar on the left middle turbinate with overlying aspergillus growth	Pale mucosa over the septum, left middle turbinate, lateral nasal wall with purulent discharge, and left middle meatus (Figure 3)	Black eschar on the left middle turbinate and lateral nasal wall (Figure 4)	Inflamed sinonasal mucosa with pus in the left middle meatus	Pale mucosa over the left middle turbinate and lateral nasal wall	Inflamed sinonasal mucosa with pus in the left middle meatus	Inflamed sinonasal mucosa with pus in the bilateral middle meatus
Disease extent	Sinonasal. Right frontal, ethmoid, maxillary sinuses	Rhino-orbital. Right frontal, ethmoid, maxillary sinuses with orbital involvement, optic neuritis, and ophthalmic artery invasion	Rhino-orbital. Left frontal, ethmoid, maxillary sinuses with orbital involvement and left hard palate	Rhino-anterior skull base. Right frontal, ethmoid, rarified superior septum, crista galli, cribriform plate, and right posterior wall of the frontal sinus	Sino-nasal. Left frontal, bilateral, ethmoid, maxillary sinuses	Rhino-orbital. Left frontal, ethmoid, maxillary sinuses and orbital involvement	Sinonasal. Left frontal, ethmoid, maxillary sinuses and left hard palate	Sino-nasal. Left frontal, ethmoid, maxillary sinuses	Rhino-orbital. Left frontal, ethmoid, maxillary sinuses and orbital involvement	Sinonasal. Bilateral frontal, ethmoid, maxillary sinuses. Progressed to bilateral, frontal, and left maxillary osteomyelitis after four weeks	Sinonasal. Bilateral frontal, ethmoid, maxillary sinuses and bilateral frontal osteomyelitis
Surgical intervention	Stage 1: ESS-debridement-right Draf IIa + external frontal sinusotomy. Stage 2: revision ESS debridement after three weeks	Stage 1: ESS-debridement-right Draf IIa + external frontal sinusotomy + orbital exploration. Stage 2: revision ESS debridement + right orbital exenteration after four weeks	ESS-debridement-left Draf IIa + external frontal sinusotomy + orbital exenteration + left infrastructure maxillectomy	ESS-debridement-right Draf IIa + external frontal sinusotomy	ESS-debridement-left Draf IIa + external frontal sinusotomy	ESS-debridement-left Draf IIa + external frontal sinusotomy + orbital exploration	Stage 1: ESS-debridement-left Draf IIa + external frontal sinusotomy + infrastructure maxillectomy. Stage 2: revision ESS debridement + left total maxillectomy after four weeks	ESS-debridement-left Draf IIa + external frontal sinusotomy	Stage 1: ESS-debridement-left Draf IIa + external frontal sinusotomy + orbital exploration. Stage 2: revision ESS debridement + orbital exenteration after five weeks	Stage 1: ESS-debridement-Draf IIa + bilateral external frontal sinusotomy. Stage 2: revision ESS debridement + left frontal empyema drainage after two weeks. Stage 3: planned for bicoronal approach-frontal sequestrectomy and left maxillectomy after six weeks (no consent from the patient)	ESS-debridement-Draf IIa + bilateral external frontal sinusotomy (no consent for bicoronal approach)
Frontal sinus endoscopy	Pus with fungal debris and inflamed mucosa in the frontal sinus (Figure 5)	Pale frontal sinus mucosa with fungal debris (Figure 6)	Pale frontal sinus mucosa with fungal debris, with necrotic black spots (Figure 7)	Dural prolapse into the right frontal sinus due to the right posterior frontal sinus wall erosion (Figure 8)	Necrotic black frontal sinus mucosa with fungal growth (Figure 9)	Pus with pale frontal sinus mucosa with fungal debris (Figure 10)	Pale frontal sinus mucosa studded with necrotic black spots (Figure 11)	Pus with fungal debris, in the frontal sinus up to the lateral corner (Figure 12)	Pale frontal sinus mucosa with fungal debris	Pus with inflamed mucosa and left frontal sinus (first surgery). Pale frontal sinus mucosa with fungal debris with granulations (second surgery)	Inflamed frontal sinus mucosa with fungal debris granulations, with posterior frontal sinus wall focal erosions (Figure 13)
Tissue culture	*Rhizopus*, aspergillus, and *Pseudomonas*	*Rhizopus*, *Klebsiella*, and *Enterobacter cloacae* complex	*Rhizopus*, aspergillus, and *Pseudomonas*	Rhizopus	*Rhizopus*, aspergillus, and *Candida*	*Rhizopus* and *Klebsiella*	*Rhizopus* and aspergillus	*Rhizopus* and *Klebsiella*	*Rhizopus* and *Pseudomonas*	*Rhizopus* and *Pseudomonas*	*Rhizopus*, *Klebsiella*, and *E. coli*
Histopathology	Tissue-invasive, angioinvasive mucormycosis	Tissue-invasive, angioinvasive mucormycosis	Tissue-invasive, angioinvasive mucormycosis	Tissue-invasive mucormycosis	Tissue-invasive, angioinvasive mucor-mycosis	Tissue-invasive, angioinvasive mucormycosis	Tissue-invasive, angioinvasive mucormycosis	Tissue-invasive mucormycosis	Tissue-invasive, angioinvasive mucormycosis	Tissue-invasive, angioinvasive mucormycosis	Tissue-invasive, angioinvasive mucormycosis
Medical management	IV liposomal amphotericin B: seven days. Oral isavuconazole: eight weeks	IV liposomal amphotericin B: 14 days. Oral posaconazole: 12 weeks	IV liposomal amphotericin B: two days	IV liposomal amphotericin B: 14 days. Oral posaconazole: 12 weeks	IV liposomal amphotericin B: five days	Posaconazole: seven days	IV liposomal amphotericin B: 14 days. Oral posaconazole: 12 weeks	IV liposomal amphotericin B: 14 days. Oral posaconazole: eight weeks	IV liposomal amphotericin B: 10 days. Oral isavuconazole: 12 weeks	IV liposomal amphotericin B: 10 days. Oral posaconazole: eight weeks	IV liposomal amphotericin B: three days
Outcome in one-year follow-up	Clear	Clear	Died: second POD. Septic shock and pulmonary thromboembolism	Nonprogressive anterior skull base findings. Clear sinonasal mucosa	Died: sixth POD. Septic shock	Died: seventh POD. Sepsis and multiorgan failure	Clear	Clear	Clear	Lost to follow-up by eight weeks	Died: fourth POD. Dyselectrolemia/sepsis/cardiac failure

**Figure 2:**
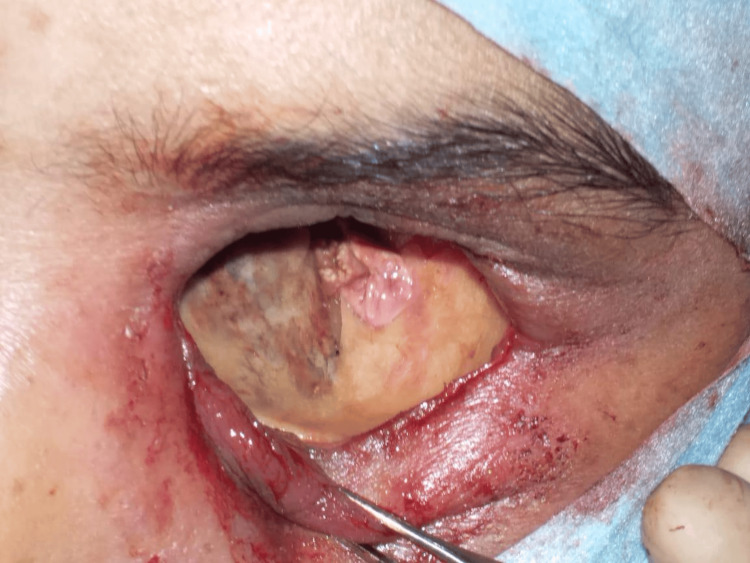
Necrotic lamina papyracea

**Figure 3:**
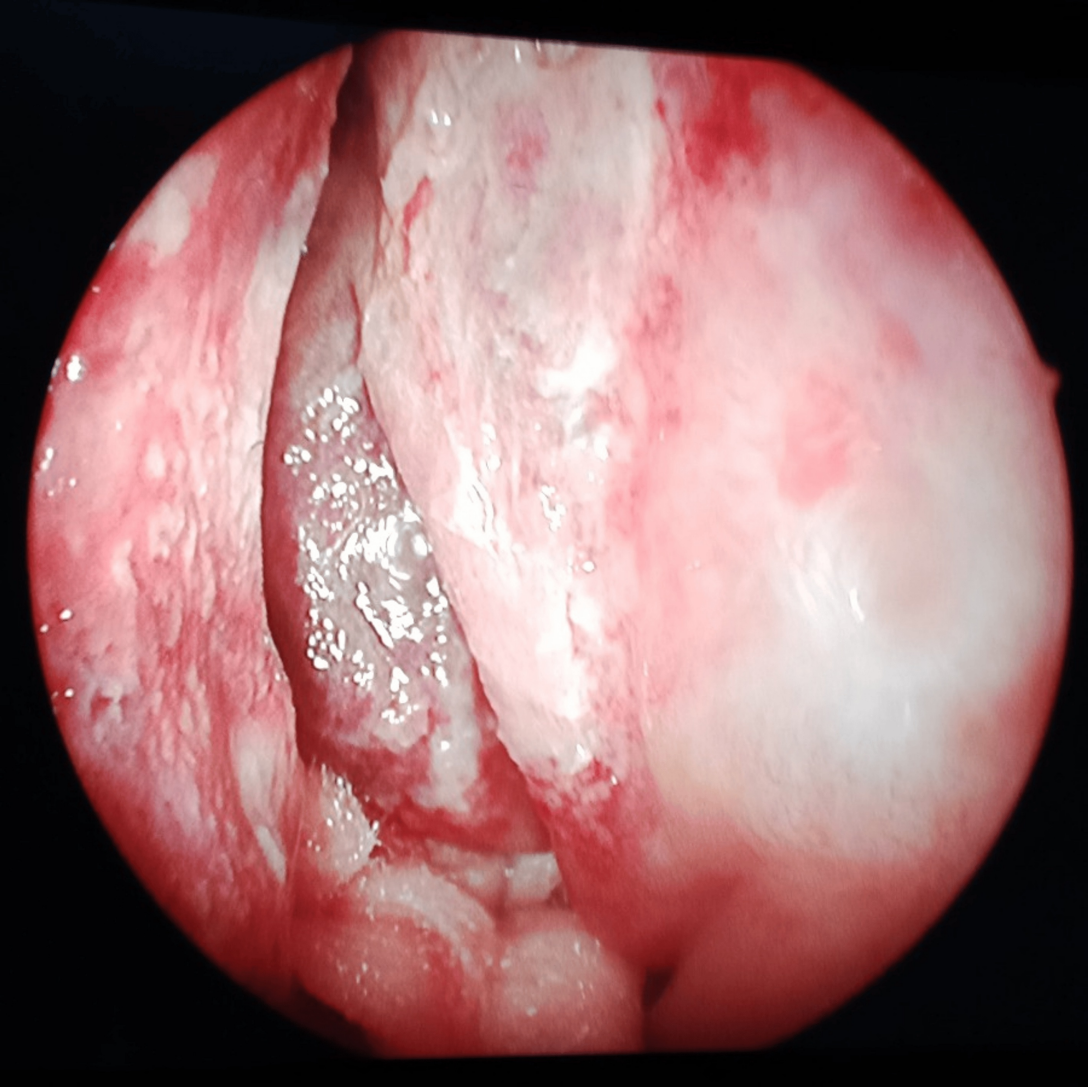
Nasal endoscopy Pale septum, middle turbinate, and lateral nasal wall

**Figure 4:**
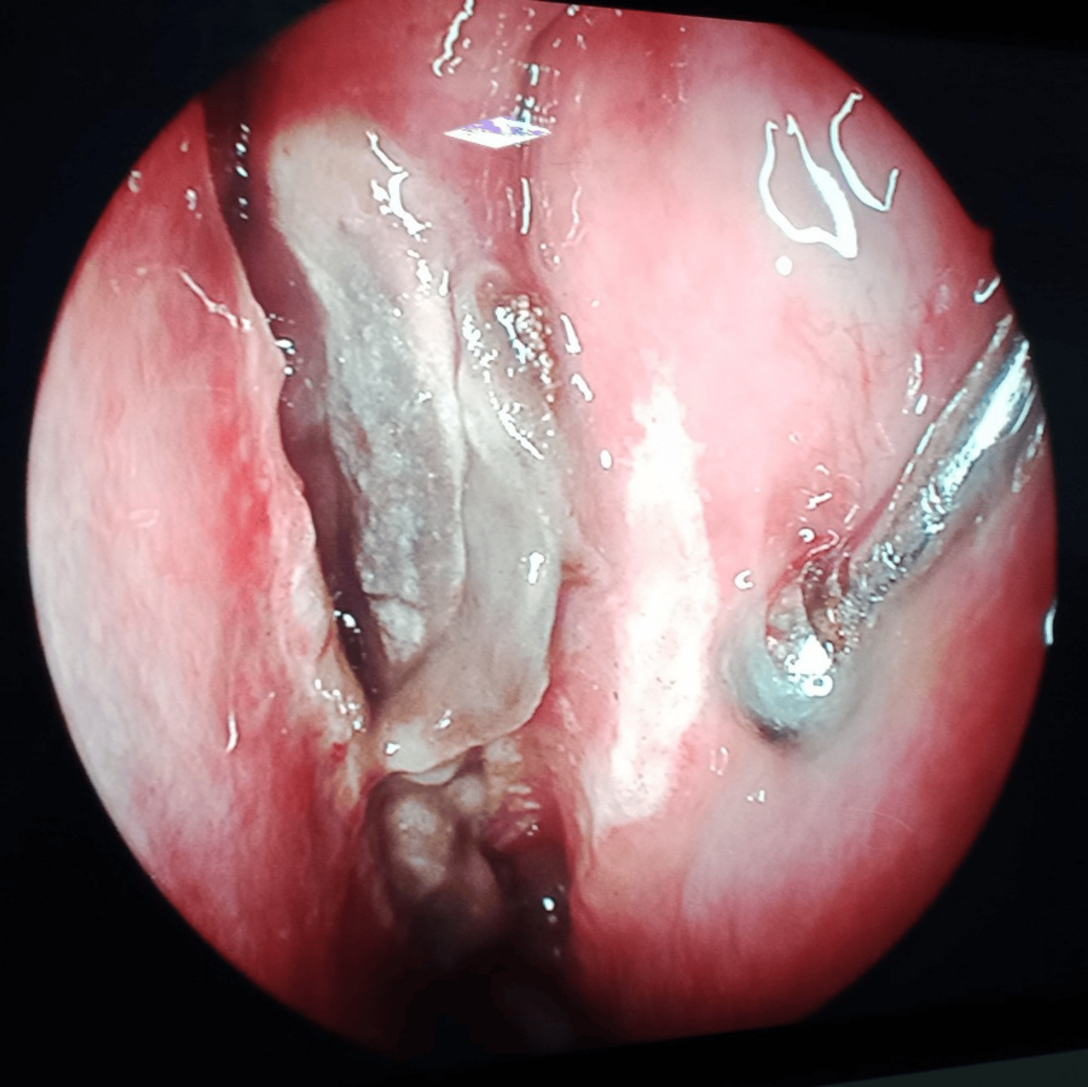
Nasal endoscopy Necrotic middle turbinate with black eschar

**Figure 5:**
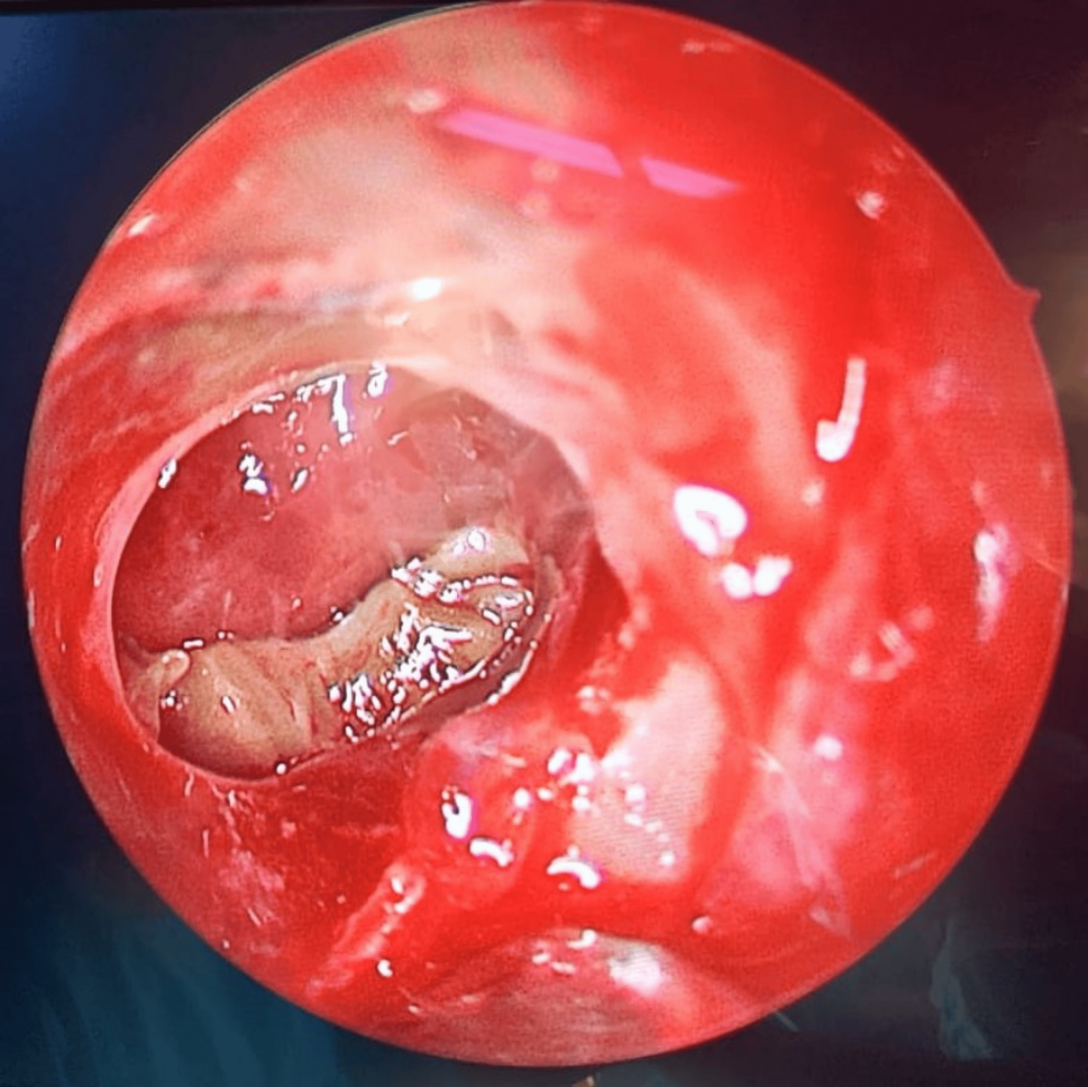
Frontal sinus endoscopy Fungal debris in the frontal sinus

**Figure 6:**
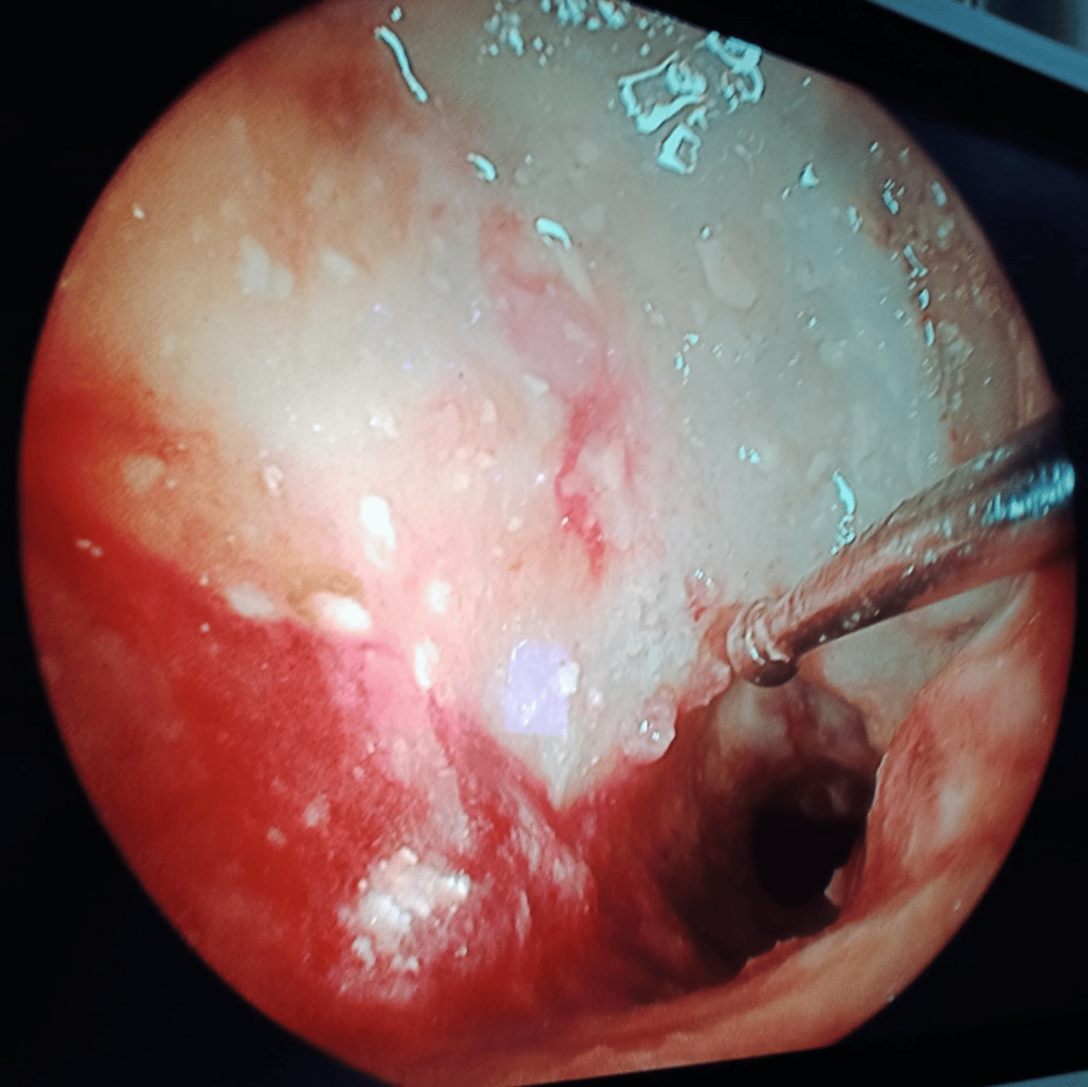
Frontal sinus endoscopy Pale mucosa with fungal debris in the frontal sinus

**Figure 7:**
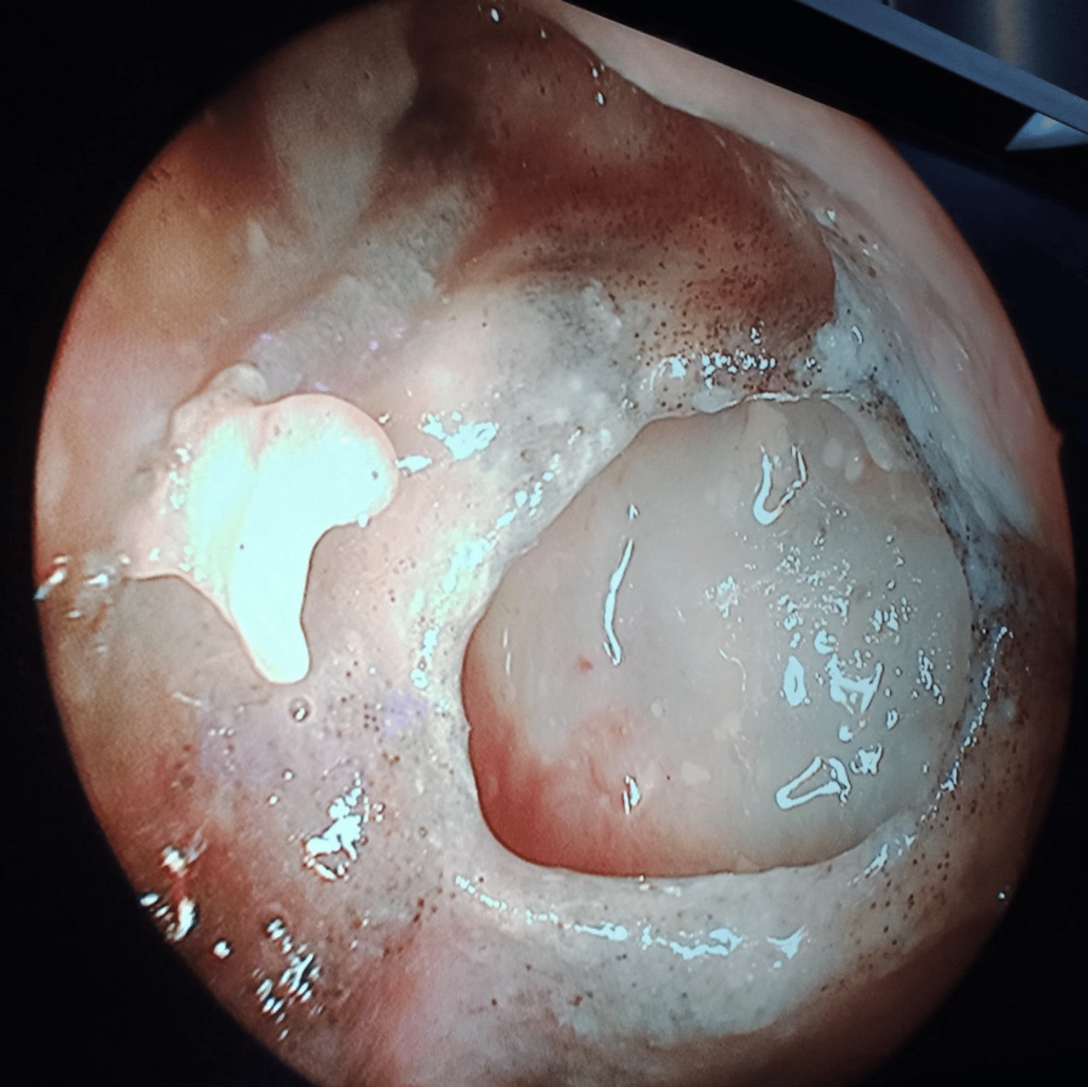
Frontal sinus endoscopy Pale mucosa with necrotic black spots in the frontal sinus

**Figure 8:**
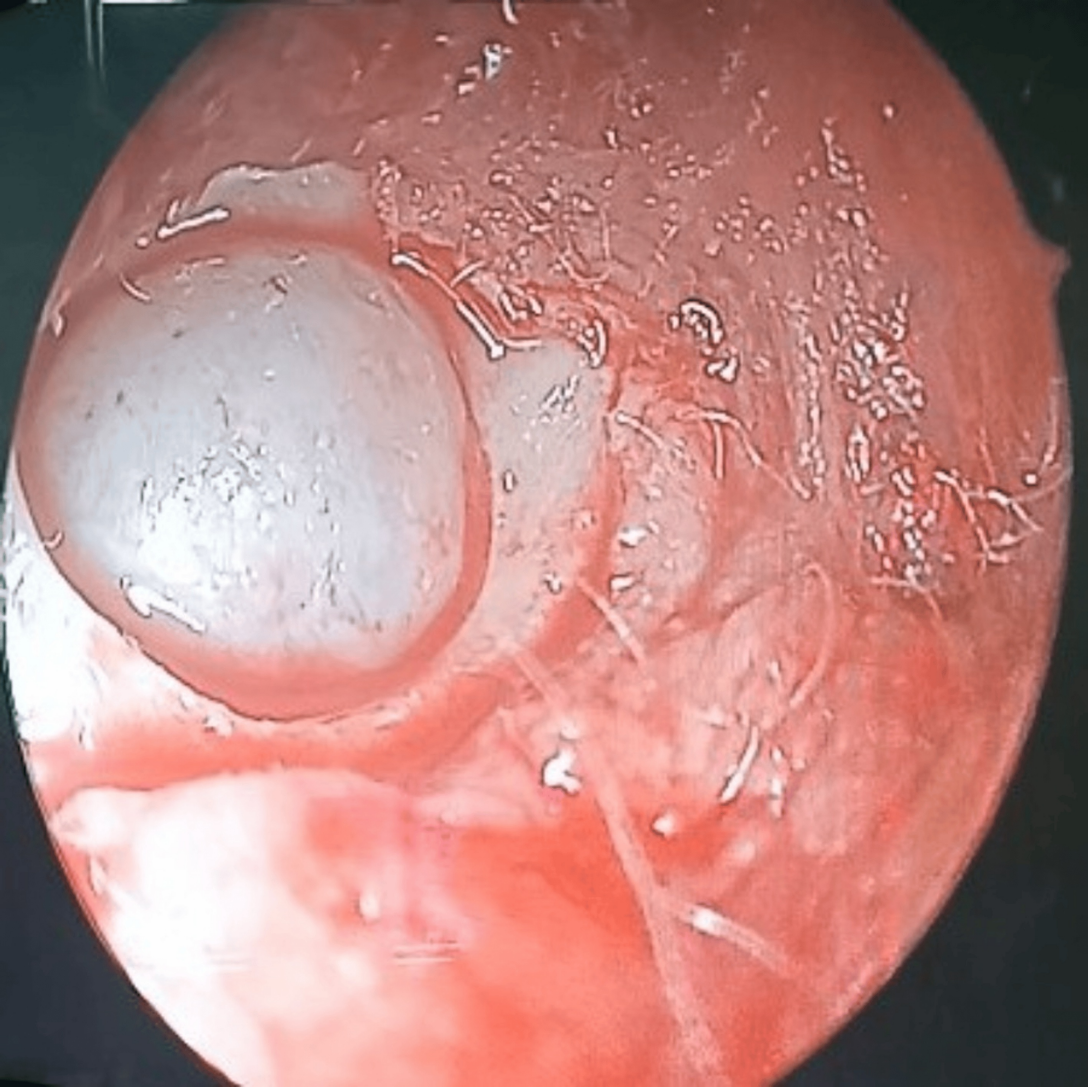
Frontal sinus endoscopy Dural prolapse into the right frontal sinus due to posterior frontal sinus wall erosion

**Figure 9:**
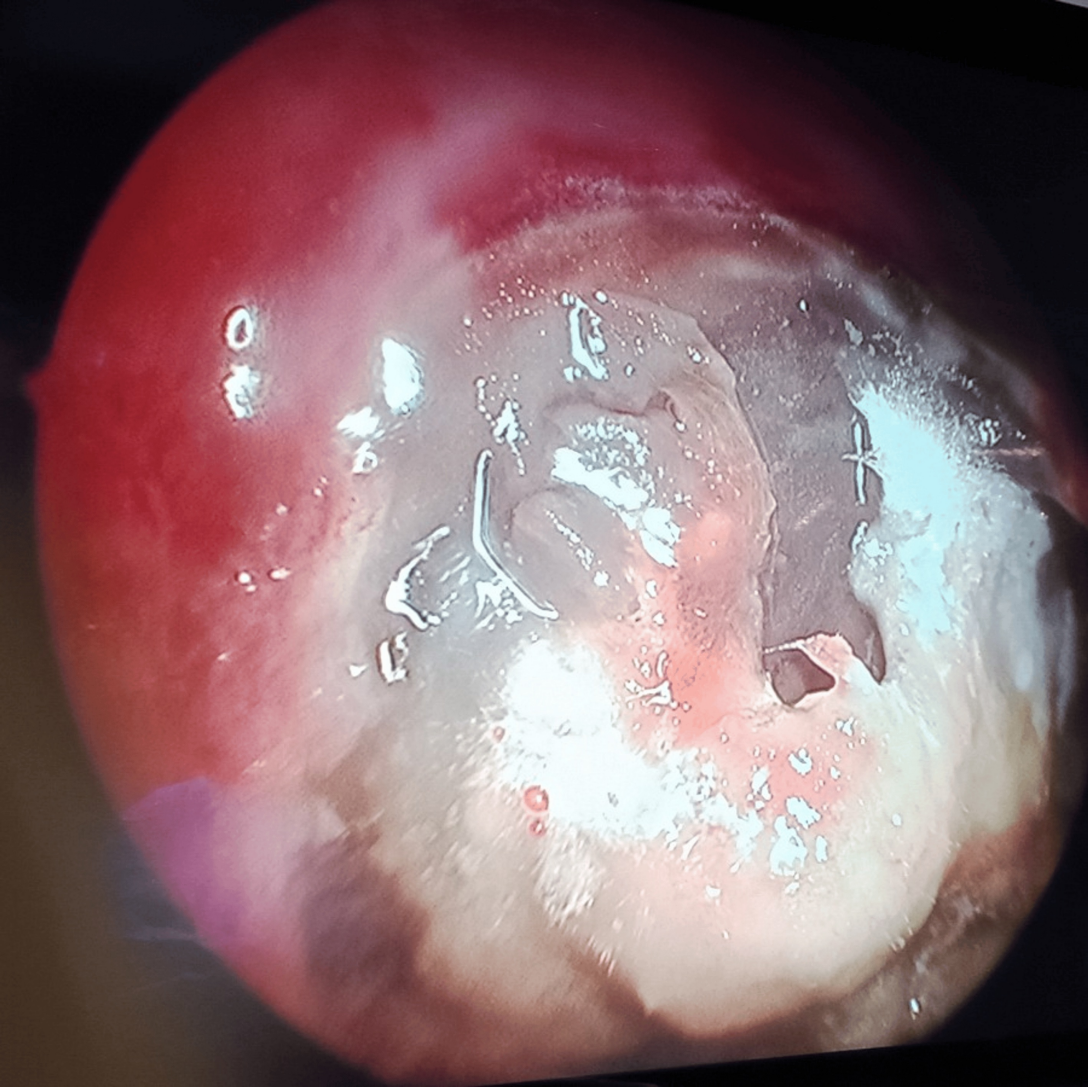
Frontal sinus endoscopy Necrotic black frontal sinus mucosa with fungal growth in the frontal sinus

**Figure 10:**
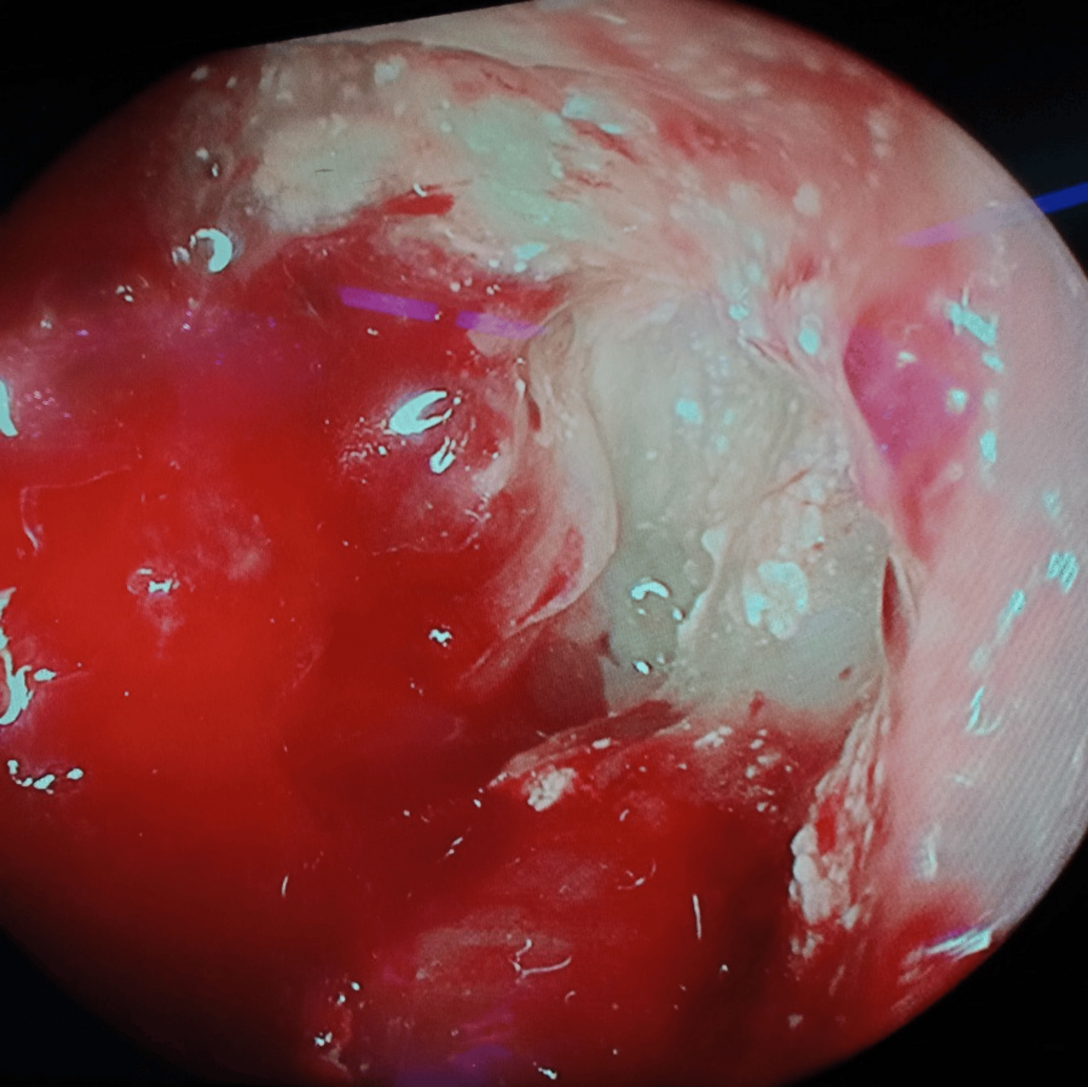
Frontal sinus endoscopy Pale mucosa with fungal debris in the lateral corner of the frontal sinus

**Figure 11:**
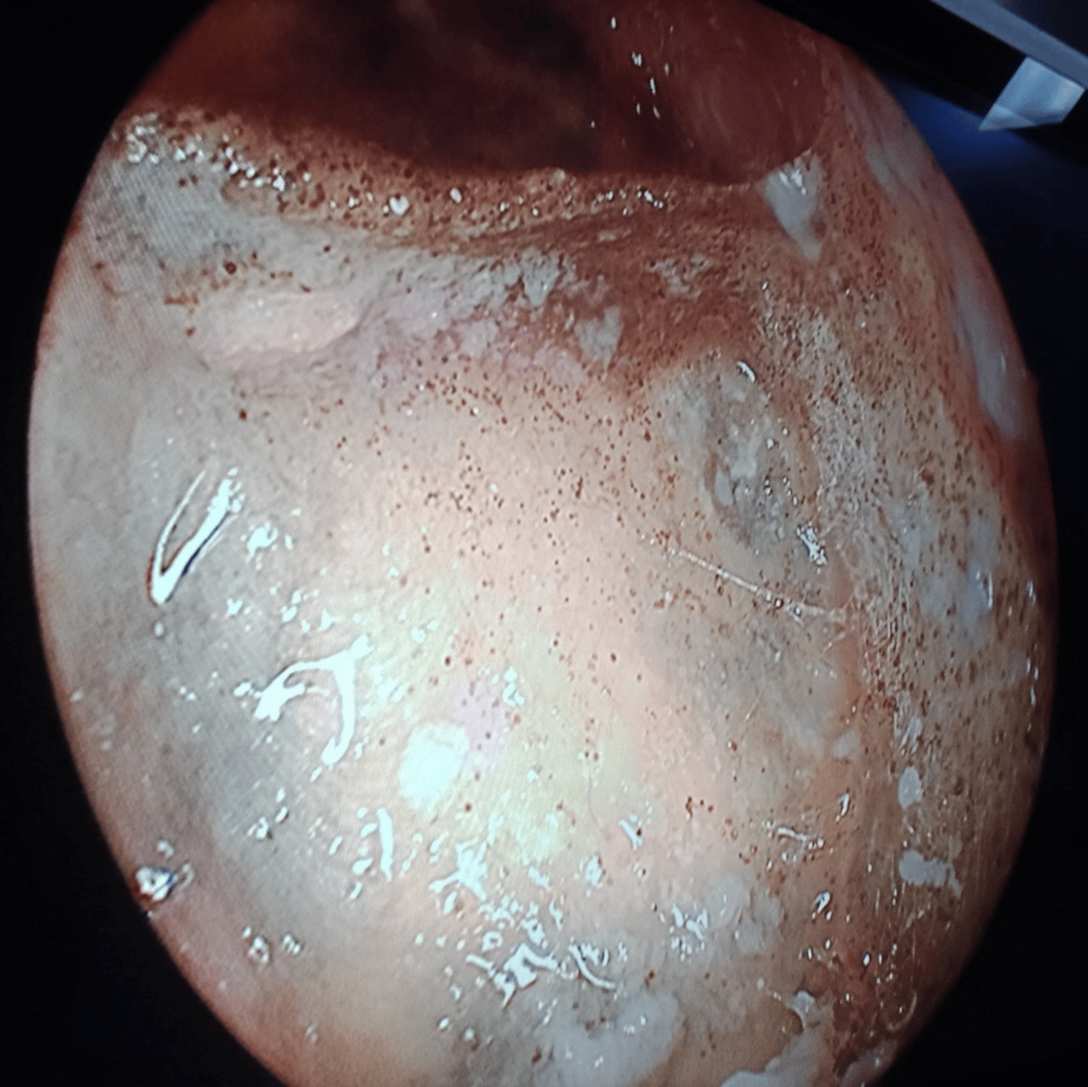
Frontal sinus endoscopy Pale frontal sinus mucosa studded with necrotic black spots

**Figure 12:**
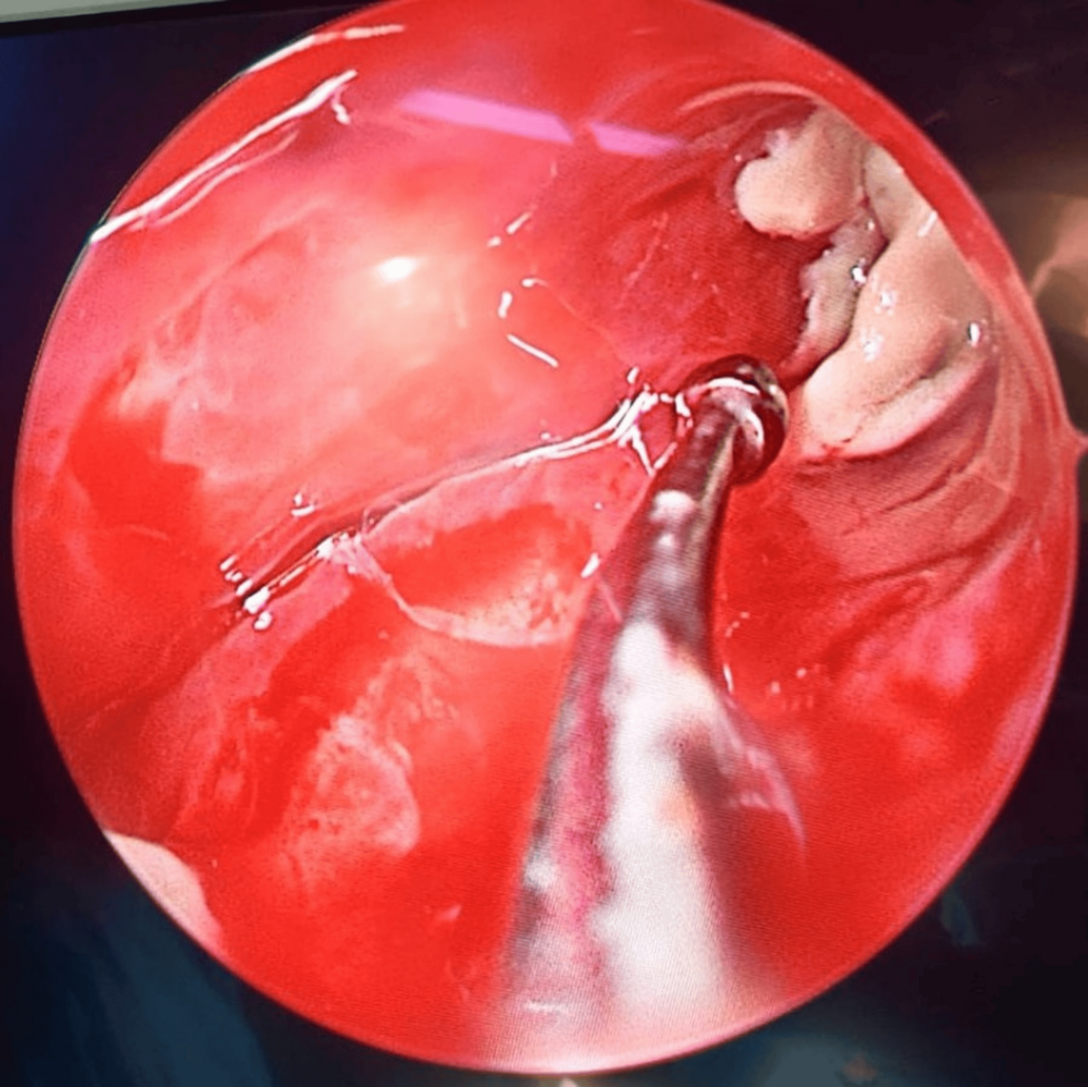
Frontal sinus endoscopy Fungal debris in the lateral corner of the frontal sinus

**Figure 13:**
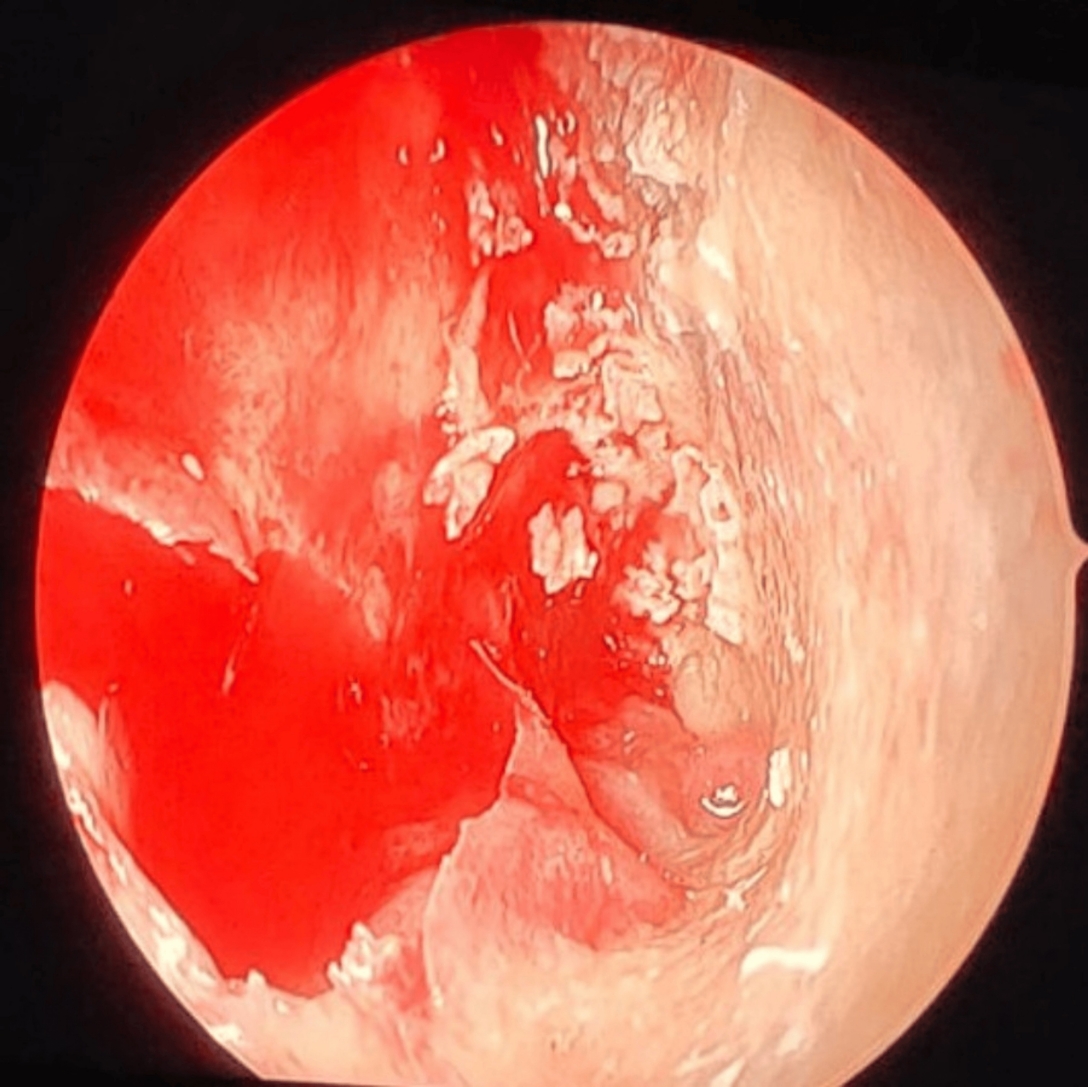
Frontal sinus endoscopy Granulations with posterior frontal sinus wall focal erosion

The most common clinical symptom was headache (91%), followed by periorbital pain and swelling (73%), though two patients (18%) had ocular pain without periorbital swelling. Other symptoms were nasal block and nasal discharge (55%), anosmia (46%), and visual symptoms (36%) with proptosis, chemosis, ophthalmoplegia, and vision loss. One patient (9%) had vision loss without proptosis, chemosis, or ophthalmoplegia. The least common symptom in this study was dental pain (18%).

Nasal endoscopy revealed the mucosal changes most predominant in the middle turbinate, followed by the lateral nasal wall and the septum. The classic black eschar was noted in five patients (46%), pale mucosa in three patients (27%), and inflamed mucosa with purulent discharge in three patients (27%). Disease extent was limited to nose and paranasal sinus mucosa in four patients (36%), rhino-orbital extension in four patients (36%), rhino-anterior skull base extension in one patient (9%), palatal eschar in two patients (18%), and frontal bone osteomyelitis in two patients (18%), and one (9%) among them had coexisting maxillary bone osteomyelitis.

All 11 patients underwent endoscopic sinus surgery: bilateral pansinus debridement involving endoscopic Draf IIa and external frontal sinusotomy (unilateral in nine patients and bilateral in two patients). Three (27%) patients with orbital involvement underwent orbital exenteration. Two patients (18%) had infrastructural maxillectomy, and one (9%) among them had a revision total maxillectomy for progressive disease. Five (46%) patients after the first surgical treatment underwent revision sinus debridement surgery.

Frontal sinus endoscopy through a medial eyebrow incision revealed necrotic black frontal sinus mucosa with fungal growth (9%); pale frontal sinus mucosa studded with necrotic black spots (18%); pale frontal sinus mucosa with fungal debris (27%); inflamed frontal sinus mucosa with pus, fungal debris (18%), and granulations (18%); and prolapsed dura into the frontal sinus due to posterior frontal sinus wall erosion (9%).

**Figure 14:**
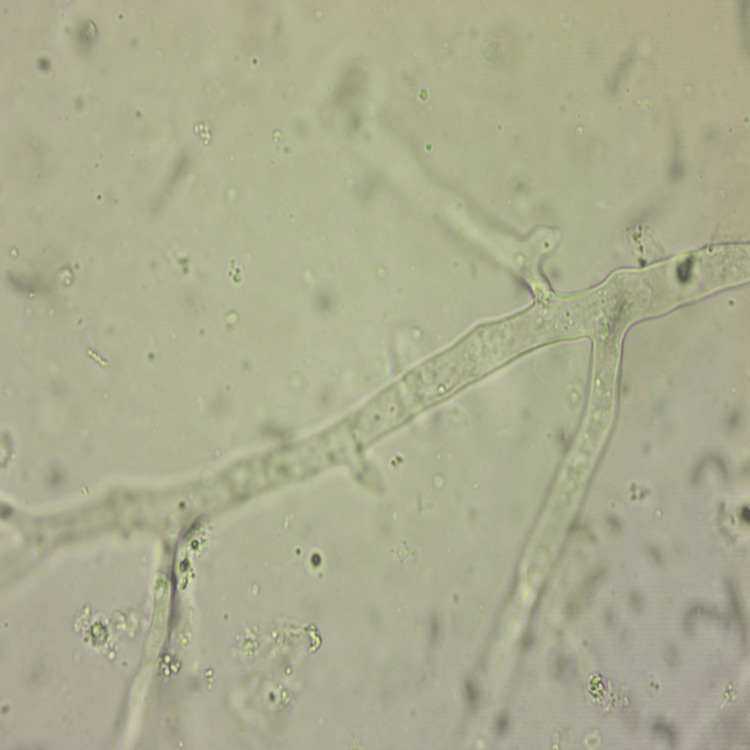
Fungal smear Ten percent potassium hydroxide (KOH) mount showing broad aseptate hyphae with wide-angle branching

**Figure 15:**
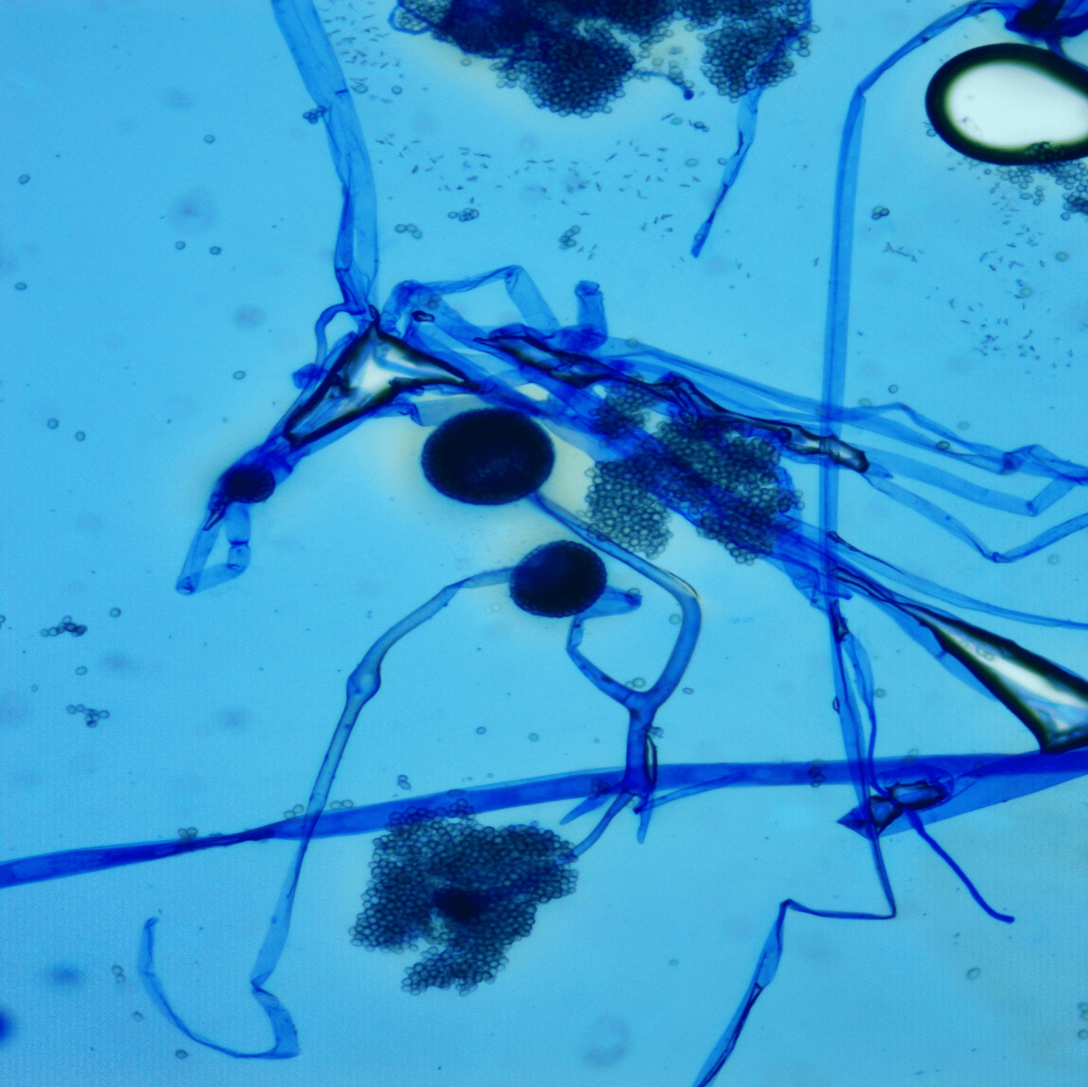
Tissue culture Lactophenol cotton blue (LPCB) mount showing *Rhizopus* species from culture isolate

**Figure 16:**
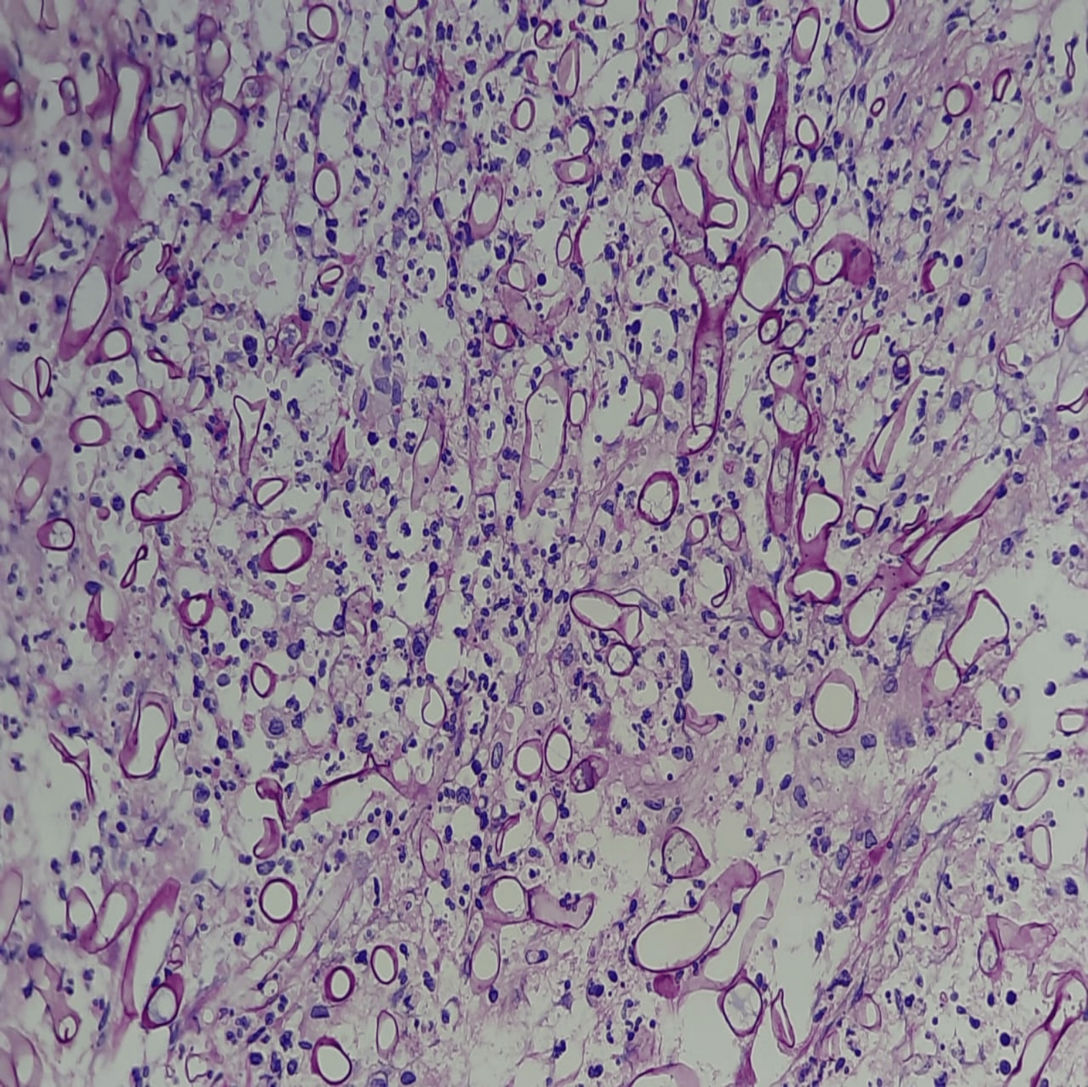
Histopathological section Periodic acid-Schiff (PAS) stain showing numerous large aseptate hyphae consistent with the morphology of mucormycosis

Operative samples for fungal smear (Figure 14) showed broad aseptate hyphae with wide-angle branching consistent with mucor, and tissue culture revealed *Rhizopus* species (Figure 15) for all 11 patients, though the coexisting growth of *Aspergillus*, *Candida*, *Pseudomonas*, *Klebsiella*, *E. coli*, and *Enterobacter* was noted. Operative tissue in histopathology (Figure 16) revealed tissue and angioinvasive mucor in nine (82%) out of 11 patients and tissue-invasive mucor in two (18%) patients. Ten (91%) out of 11 patients had intravenous liposomal amphotericin B (3-5 mg/kg/day) for a period of 2-14 days based on their tolerance to the drug and survival. One (9%) patient with chronic kidney disease undergoing hemodialysis was started on posaconazole 300 mg intravenous twice daily on day 1, followed by 300 mg/day for seven days. Maintenance therapy with oral posaconazole (300 mg/day for 6-12 weeks) was administered for five (46%) patients and oral isavuconazole (200 mg twice daily for 8-12 weeks) for two (18%) patients who showed prolonged QT interval in ECG after posaconazole. Six (55%) out of 11 patients recovered and were declared cured from the disease. Four (36%) out of 11 patients died within the first week of stage 1 surgical debridement. One patient (9%) was lost to follow-up after eight weeks of stage 1 surgical debridement and did not consent to the bicoronal approach and total maxillectomy toward the progressing bilateral frontal and left maxillary osteomyelitis.

## Discussion

In the present study, frontal sinus endoscopy documented varied presentations of invasive frontal sinus mucormycosis, such as necrotic black frontal sinus mucosa; pale frontal sinus mucosa studded with necrotic black spots; inflamed frontal sinus mucosa with pus, fungal debris, and granulations; and prolapsed dura into the frontal sinus due to posterior frontal sinus wall erosion. Of the patients, 55% recovered from the disease. The combined approach involving endoscopic and external frontal sinusotomy provided complete local surgical clearance up to the lateral corners and the walls of the frontal sinuses, which are nasal endoscopically inaccessible.

Sinonasal mucormycosis is caused by a saprophytic fungus belonging to the Phycomycetes class, Mucorales order, and Mucoraceae family of genus *Mucor* and *Rhizopus* [4]. The spores are widely spread in the soil, air, decaying organic material, and bed linen in medical hospitals and as a commensal in the nasal mucosa of healthy people. These spores can lodge and germinate in the nasal mucosa of immunocompromised patients [5].

The fulminant inflammation due to COVID-19, the excessive use of steroids for COVID-19-induced complications, and preexisting complications such as diabetes dysregulate the immune response, potentially increasing the risk of developing fungal co-infections. COVID-19 causes the overexpression of inflammatory cytokines and reduces cluster of differentiation 4+ (CD4+) T cell and CD8+ T cell counts, thus impairing cell-mediated immunity, paving the way for opportunistic fungal infections [6]. Three patients in this study had the onset of sinonasal symptoms suggestive of fungal sinusitis within seven days of COVID-19. They were observed to have high morbidity and mortality due to sepsis, which could be explained by the rapidly progressing invasive disease coexisting with fulminant inflammation due to COVID-19.

Uncontrolled diabetes provides a high-glucose, low-oxygen acidic environment with deranged granulocyte phagocytic activity, altered leucocyte response, and microangiopathy, resulting in local tissue ischemia facilitating the growth of fungal infections [7]. We observed diabetes to be a significant comorbidity associated with eight out of 11 patients. Further, coexisting multiple comorbidities such as coronary artery disease, chronic liver and kidney disease, and chronic obstructive pulmonary disease add morbidity for the patients irrespective of their age, sex, and steroid therapy for COVID-19.

The common symptoms of sinonasal mucormycosis are headache, facial pain, nasal discharge, epistaxis, numbness over the cheek, fever, periorbital swelling, vision disturbances, proptosis, orbital cellulitis, ophthalmoplegia, altered mental status, and seizures as per the extent of the disease [8]. Even in this present study, headache and facial/periorbital pain were the predominant symptoms.

Mucorales are angioinvasive, invading the arteries and causing extensive endothelial damage and subsequently arterial thrombosis, resulting in tissue ischemia and leading to gangrene and the classic black necrotic eschar formation [9]. Nasal endoscopy in post-COVID-19 patients with sinonasal symptoms can pick up early signs of mucormycosis well before the imaging studies. The classic black necrotic eschar, or pale mucosa with purulent discharge, needs an immediate tissue biopsy and fungal smear for prompt diagnosis and to proceed with the treatment. Aseptate broad hyphae with branching at right angles with tissue and angioinvasion in histopathology are characteristics of invasive mucormycosis.

The predominantly involved sites include the middle turbinate (67%), septum (21%), palate (19%), and inferior turbinate (10%) [10]. The most commonly affected sinuses are ethmoids, followed by maxillary, sphenoid, and frontal sinuses [11]. The frontal sinus is the least commonly affected sinus as the ostium is located in the anterosuperior part of the nasal cavity, making it less feasible for the lodgment of the fungal spores [12]. In the above study, the middle turbinate was the most commonly affected site, and the ethmoid was the commonly affected sinus, suggesting the spread of the disease from the ethmoid to the frontal sinus. Though nasal endoscopy is the prime diagnostic test for mucormycosis, a CT scan of the nose and paranasal sinuses, including the orbit and brain, is needed for assessing the extent of the disease, surgical planning, and postoperative planning as needed. The MRI of paranasal sinuses with the orbit and brain helps to assess the disease with orbital and intracranial involvement [4].

This invasive, potentially fatal disease has a high mortality rate of 50%-80% in untreated patients [13]. As per the recommendations of “Global guidelines for the diagnosis and management of mucormycosis 2019,” early diagnosis and prompt management with appropriate imaging, surgical intervention, and intravenous liposomal amphotericin B are strongly recommended [14]. Treatment involves a multidisciplinary approach involving early diagnosis, prompt surgical debridement, managing the underlying comorbidities, IV antifungal therapy, and maintenance therapy with oral antifungals, followed by close regular follow-up for a minimum period of one year with nasal endoscopy and imaging as required.

Intravenous amphotericin and oral antifungals have nil access to the infarcted necrosed tissues. Extensive debridement until reaching fresh bleeding healthy tissues and tissue biopsy from the margins of debridement are absolutely necessary to achieve a good local disease clearance and to define the extent of debridement. Intravenous liposomal amphotericin B (3-5 mg/kg/day) is the first-line drug of choice for a period of 14 days, depending on the patients’ tolerance toward the drug; stepping down to maintenance therapy with oral posaconazole or isavuconazole for a period of 6-12 weeks based on the clinical course of healing is mandatory [3]. In the present study, 10 out of 11 patients had intravenous liposomal amphotericin B as the first-line drug. Maintenance therapy with oral posaconazole was administered for 6-12 weeks, and oral isavuconazole was administered for the patients who developed prolonged QT interval following posaconazole therapy. Close regular follow-up is crucial in the first three months, extending up to one year to pick up recurrences, new development of disease at new sites, and progression to osteomyelitis requiring revision surgeries.

Related to our special focus on the frontal sinus involvement, endoscopically, the Messerklinger technique adopted in the functional endoscopic sinus surgery (FESS) involves disease clearance in the frontal recess, with mucosal preservation and widening of the frontonasal duct pathway, thereby restoring the physiological mucociliary clearance pattern. The lateral corners and the walls of the frontal sinuses are inaccessible endoscopically and do not require any surgical work in regular FESS. Uncommonly invasive fungal frontal sinusitis due to mucormycosis has a dreadful, rapid, progressive nature crossing the bony boundaries, causing significant necrosis and the extension of the disease, involving frontal sinus mucosal necrosis and bony wall erosion with debris extending up to the lateral corners of the frontal sinuses. Hence, complete local disease clearance by extensive debridement of the necrosed tissue is vital to halt the disease progression. This raises the necessity of combining external with an endoscopic approach targeting complete local disease clearance [12]. Both these techniques are proven existing techniques adopted individually for various indications. External frontal sinus surgeries such as trephination, external frontal sinusotomy, and osteoplastic flap surgeries are adopted for frontal abscess, mucocele, frontal sinus wall trauma, tumor, osteomyelitis, etc. [15,16]. Among these, osteoplastic flap surgeries are considered to be the principal method for frontal sinus access before the advent of endoscopes, which is more invasive and involves neurosurgical association [17].

In the endoscopic era, minimally invasive frontal sinus external approaches combined with angled endoscopic guidance can provide good disease clearance. Frontal trephination was combined with endoscopic frontal sinusotomy in cases of chronic recurrent frontal sinusitis with neo-osteogenesis involving the frontal recess, tumor of the frontal sinus, and mucoceles [18]. Further, combined trephination with endoscopic frontal sinusotomy was adopted in conditions with type 4 frontal cell and remote lateral frontal sinus lesions that were otherwise inaccessible endoscopically [19,20]. Combined external frontal sinusotomy with endoscopic guidance and endonasal approaches was adopted for managing CSF leak from the fractured posterior table of the frontal sinus, frontal osteoma, and lateral frontal mucocele [21]. However, extensive frontal sinus osteomyelitis, bilateral involvement requiring large areas of sequestrum removal with or without mesh reconstruction, and revision surgeries after multiple failed endonasal attempts require more invasive osteoplastic flap surgeries through bicoronal incisions [22].

We adopted, modified Lynch Howarth medial eyebrow incision (1.5 cm medial eyebrow incision, followed by the dissection of orbicularis oculi muscle and the drilling of 1-1.5 cm-width frontal sinusotomy) for external frontal sinusotomy combined with endoscopic Draf IIa for all 11 patients with invasive mucormycosis, which helped in achieving complete local disease clearance. The grey areas of the frontal sinuses (lateral corners and the walls of the frontal sinuses) in these invasive mucormycosis are addressed by the external frontal sinusotomy combined with angled endoscopic guidance; thereby, complete local disease clearance was made possible.

Further, frontal sinus endoscopy with angled endoscopes showing the necrotic black frontal sinus mucosa with fungal growth, pale mucosa with studded necrotic black spots, and fungal debris extending until the lateral corners explains the necessity of combining external with endoscopic approaches for frontal sinus involvement in invasive mucormycosis. As per our experience, well evident from the frontal sinus endoscopy, sinonasal invasive fungal disease, especially mucormycosis involving the frontal sinuses, needs combining an external approach with endoscopic access to achieve complete disease clearance.

## Conclusions

Frontal sinus endoscopic findings explain the necessity of combining external with endoscopic approaches for frontal sinus involvement in invasive mucormycosis. The endoscopic findings in invasive frontal mucormycosis are documented in this article for the first time, which add a significant input to the existing medical literature.

Though the endoscopic approach for frontal sinusitis is adequate for resuming the mucociliary clearance in the regular functional endoscopic frontal sinus surgery, for invasive sinonasal mucormycosis involving frontal sinuses, combining with external frontal procedures provides complete local disease clearance, which is otherwise inaccessible.
